# Advancing reuse of genetic parts: progress and remaining challenges

**DOI:** 10.1038/s41467-023-38791-0

**Published:** 2023-05-23

**Authors:** Jeanet Mante, Chris J. Myers

**Affiliations:** Department of Electrical, Computer, and Energy Engineering University of Colorado, Boulder, CO 80309 USA

**Keywords:** Data publication and archiving, Standards

## Abstract

Issues with data reuse have been recognized in synthetic biology and the broader scientific community. Policies and standards fall short as machine reasoning is not emphasised and enforcement is lacking. We discuss the progress, remaining challenges, and possible solutions.

Twelve years ago, a letter was written to highlight the lack of reproducibility and reuse in synthetic biology due to the scarcity of sequence data in publications^1^. This reflects the growing recognition of the critical role that data plays in advancing scientific research, innovation, and economic development. This realization has led to increased investment in data science and infrastructure, as well as greater awareness of the need for effective data management and sharing practices; data must be findable, accessible, interoperable, and reusable (FAIR)^2^, and it must be curated to achieve these goals. In synthetic biology, progress has been made on several fronts; however, there is still a ways to go to address the lack of reuse of genetic parts.

## Progress

The progress in genetic data reuse has been driven by increasing data awareness among different communities, including universities, companies, journals, and funding agencies. Following general trends in data science, these communities are taking steps to improve the standardization and storage of genetic data. To achieve this, they are implementing various policies that aim to ensure that genetic data is managed and shared in an organized manner. One of the most notable examples of these policies is the UNESCO Recommendation on Open Science, which sets guidelines for open science practices, including data storage, standardization, and accessibility. Another example is the requirement of data management plans by funding agencies, such as NSF, the CDC, NIH, and BBSRC, which ensure that data is properly stored and managed. Additionally, some journals now have requirements and/or recommendations for sequence submissions, such as Nature and Science. On the one hand, these policies are good as they have a broad scope, including genetic parts. On the other hand, the breadth leads to uncertainty about how policies should be implemented and how they should be incentivized or enforced.

Public awareness has also led to the formation and growth of community standards. Synthetic biology community standards include the Synthetic Biology Open Language (SBOL)^3^, the Standard European Vector Architecture (SEVA)^4^, and BioBrick Standards^5^. Different data standards serve different purposes. Some standards focus on the format and structure of data, others on visualization, and still others on assembly. However, all data standards serve a common goal: to promote data reuse. Establishing a clear and consistent framework for organizing, sharing, and using data standards would help to ensure that data is accessible and usable by a wide range of individuals and organizations. By working together, these standards create a robust and flexible infrastructure that supports the growth of synthetic biology.

## Remaining challenges

The progress in the field of synthetic biology mirrors the overall progress and advancements in data science. This is because many of the challenges and issues faced in synthetic biology are similar to those faced in the broader field of data science. The management of sequence data is currently facing several issues in terms of findability, accessibility, interoperability, and reusability. Whilst policies and standards theoretically address these issues, many policies are vague, do not currently address machine reasoning over data, or are not sufficiently enforced.

We envision a future where it is possible to ask a database questions like: “what are the strongest promoters to use in *Sorangiineae bacterium*?" and the database will provide a list of results that can then be filtered on further criteria such as exclusion of unwanted enzymatic restriction sites and thermal stability. Additionally, if there are limited results, the database can return alternative query suggestions like: “no results found for *Sorangiineae bacterium*, would you like to search over *Myxococcales* instead?". Once a result is opened, the page should have sufficient information to determine whether the part will work for the desired application. In the case of the *S. bacterium* promoters, it may report the relative promoter units (RPUs)^6^ measured under different environmental conditions with citations to the relevant experimental literature. While it is currently possible to answer these questions, it is by no means easy and the time and effort required deters people and wastes funding. While this may seem far-fetched, it is an attainable goal with many of the pieces already in place. The remaining hurdles are discussed below.

### Findability

Genetic parts are often difficult to locate due to the inability of machines to reason over the data and the absence of a centralized database for sequences. While databases like GenBank^7^, SynBioHub^8^, JBEI-ICE^9^, the iGEM BioBrick Registry^10^, and Addgene^11^ exist, the kinds of queries that can be run over the databases is limited both by database interfaces, what metadata is stored in the database, and data being put into the database. Some journals have clear guidelines for sequence submission backed up by a checklist for reviewers that requires verification of sequence deposition. Other journals have more hidden policies that reviewers are not required to verify. Thus, while the submission of sequence data has increased, it is by no means universal. Additionally, the metadata fields vary between the databases. For example, Addgene has information about growth in bacteria which GenBank does not. No database collects all the metadata required by the Minimum Information about Genomes Standard (MIGS)^12^. This issue may be addressed by well-indexed distributed data stores, or by a well-curated central database.

### Accessibility

The current system is plagued by data being inaccessible to humans and computers. The common practice of “data on request” is often met with a lack of response from authors^13^. Even if the data is available, it may not be available in a machine-readable format. For example,^14^ shows that most of the sequence supplementals found were in PDF format. This makes it difficult, if not impossible, for a machine to extract sequences and perform annotation or other analysis on them. To tackle this issue, it is essential that sequence data is made available not only to humans but also to machines via centralized databases that enforce standards that allow machine reasoning (i.e., machine-accessible formats). Some, but not all, metadata is already machine-accessible. For example, Genbank provides Taxonomy IDs to ground species terms. However, Addgene species are free text. Additionally, all databases could increase the use of unique identifiers and ontologies, e.g., ORCID, gene ontology^15^, sequence ontology^16^, and DOIs. Collecting broader ranges of metadata increases the number of fields that users can search and filter over. Using ontologies, allows computer reasoning (such as suggesting sub or super groups to narrow or broaden the search). Finally, using unique identifiers allows integration between different databases (e.g. looking for journal articles by the same author, or linking Uniprot^17^ and Genbank records)^18^. Alternatively, the rise of large language models (LLM), like ChatGPT, may increase the types of data that is machine accessible. However, the required information must still be present, regardless of the format. Additionally, because LLMs are not explainable machine learning, models must be very carefully evaluated before being trusted as part of the research process. To this end, a biological equivalent to the TruthfulQA benchmark will be required.

### Interoperability

The lack of sufficient metadata associated with genetic parts hinders their integration with other parts. For example, sequences often do not have metadata about enzymatic restriction sites. This is especially problematic when only partial sequence information, such as primers and references to plasmids, is available. However, even where sequences are available, the time required to run individual plasmid annotations is an unnecessary burden on researchers. If restriction site annotations were carried out during submission, researchers could easily filter out plasmids or constructs with unwanted restriction sites as part of their initial search. Ensuring full sequences are available is a good start, but we suggest also requiring the collection of metadata that covers a range of interoperability questions. The list of required metadata could be based on QUEEN (framework to generate quinable and efficiently editable nucleotide sequence resources), which is a machine-accessible framework for describing DNA construction protocols^19^.

### Reusability

There is often insufficient information to allow the reuse of sequences in new contexts. There are minimum information standards, such as those described by ref. ^20^; however, their use is still limited and enforcement is sparse. Additionally, how current genetic minimum information standards perform in the context of synthetic biology is unclear. There is limited data about the information required to predict sequence function in new organisms or in different environmental contexts. Defining what information is required for such predictions is necessary. Once this is done, the standard must be implemented in a manner compatible with the solutions discussed regarding findability, accessibility, and interoperability. Not all the information required by a minimum information standard needs to be stored in a single database; however, it must be linked in a manner that makes it possible to query the full information set. This will not only improve the FAIRness of sequence data, but also reduce the time and resources spent on duplicate characterization experiments and bioinformatics analyses, making the design and construction of synthetic constructs easier and more cost-effective.

## Conclusions

We attempted to implement the bulk of the proposed solutions in a post-hoc manner for the articles submitted to ACS Synthetic Biology^14^. However, this proved challenging due to the lack of machine-readable sequences, the difficulty of natural language processing, and the inherent ambiguity of language. Ambiguity is illustrated by the fact that *S. aureus* may be several different species, including *Scleropages aureus*, *Senecio aureus*, *Sericulus aureus*, *Somatogyrus aureus*, or *Staphylococcus aureus*. Which species is meant can sometimes, but not always, be understood from context. Instead, we suggest integrated curation that prompts authors to submit the required sequence data in machine-accessible formats with specific tags that contain grounded keywords^14,21^. The curation process could be semi-automated, and it could be part of the paper submission workflow. This would minimize the additional work required of the author. Making sequence data curation part of the submission and review process would help enforce data management policies and increase the FAIRness of sequence data. This will have a positive impact on the entire research community and make data-driven discoveries easier and more efficient.
